# Effects of Chemotherapy Treatment on Muscle Strength, Quality of Life, Fatigue, and Anxiety in Women with Breast Cancer

**DOI:** 10.3390/ijerph17197289

**Published:** 2020-10-06

**Authors:** Vitor A. Marques, João B. Ferreira-Junior, Thiago V. Lemos, Rafael F. Moraes, José Roberto de S. Junior, Rafael R. Alves, Maria S. Silva, Ruffo de Freitas-Junior, Carlos A. Vieira

**Affiliations:** 1 Postgraduate Program in Health Sciences, Federal University of Goias, Goiania 74605-050, GO, Brazil; rafaelfmmoraes@hotmail.com (R.F.M.); alves.rafael.ribeiro@gmail.com (R.R.A.); maria2593857@hotmail.com (M.S.S.); vieiraca11@gmail.com (C.A.V.); 2 Federal Institute of Sudeste of Minas Gerais- Campus Rio Pomba, Rio Pomba 36180-000, MG, Brazil; jbfjunior@gmail.com; 3 School of Physical Education and Physiotherapy, State University of Goias, Goiania 74643-010, GO, Brazil; tvlemos@gmail.com; 4 Teacher and Humanities Training School, Pontifical Catholic University of Goias, Goiania 74605-010, GO, Brazil; 5 Postgraduation Program in Sciences and Technologies in Health, University of Brasília, Brasília 72220-275, DF, Brazil; joserobertofisio@gmail.com; 6 Advanced Center for Diagnosis of Breast Cancer (CORA/HC/UFG/EBSERH), Clinical Hospital, Federal University of Goias, Goiania 74605-050, GO, Brazil; ruffojr@terra.com.br; 7 School of Physical Education and Dance, Federal University of Goias, Goiania 74690-900, GO, Brazil

**Keywords:** isokinetic test, isometric strength, physical exercise, physical activity, psychobiological profile

## Abstract

The study aimed to evaluate the effects of chemotherapy treatment on muscle strength, quality of life, fatigue, and anxiety in women with breast cancer. Nineteen women who were undergoing a chemotherapy treatment (breast cancer treatment [BCT] group, 52.2 ± 13.1 years) and 18 women without cancer (control [CNT] group, 55.8 ± 8.4 years) answered questionnaires for evaluation of fatigue (Fatigue Scale), quality of life (Short-Form Healthy Survey [SF-36] questionnaire), and anxiety (State-Trait Anxiety Inventory [IDATE]) levels. Muscle strength was also assessed by an isometric grip test and an isokinetic knee extension test. Physical limitations, social and emotional domains of quality of life were lower in the BCT group in comparison to the CNT group (*p* = 0.002; *p* = 0.003; *p* = 0.0003, respectively). The other domains did not differ between groups (*p* > 0.05). There were no differences in fatigue and anxiety levels between both the BCT and CNT groups (*p* > 0.05). Additionally, isometric grip strength was higher in the CNT group when compared to the BCT group (*p* = 0.048). However, there were no differences between the BCT and CNT groups for peak torque and total work at both 60°.s^−1^ (*p* = 0.95 and *p* = 0.61, respectively) and 180°.s^−1^ (*p* = 0.94 and *p* = 0.72, respectively). These results suggest that three cycles of chemotherapy treatment may impair handgrip isometric strength and quality of life in women with breast cancer.

## 1. Introduction

Cancer has been considered a public health problem nearly all over the world, especially in developed countries [1]. More than 18 million new cases were registered in 2018, and more than nine million people died from cancer worldwide [1]. Among the several types of cancer, breast cancer can be highlighted as the main cause of death by cancer in women. In 2018, approximately 2.089 million new cases of breast cancer were detected, representing 24.2% of all cancer types diagnosed in women [1].

An option for breast cancer treatment is chemotherapy, which may bring adverse effects such as impairment in quality of life and fatigue. Quality of life is related to subjective parameters usually linked to health conditions and factors that may affect the subject’s capacity of living a full life [2]. Cancer-related fatigue is a very common side effect caused by chemotherapy, and it is normally linked to pain, sleep disorders, anemia, and cachexia [3]. As a result, chemotherapy treatment brings serious damage to women with breast cancer, reducing their functional capacity, quality of life, and life expectancy [4]. All these factors may cause a reduction in overall physical activity levels, leading to a downward spiral of reduced muscle performance and worsening of fatigue and quality of life [3,4].

It is well known that an adequate level of muscle strength is required to complete daily living activities, and lower levels of muscular strength seem to be associated with greater rates of mortality by cancer [5,6]. It has also been shown that cancer-related fatigue was negatively associated with muscle strength [7,8]. Additionally, the chemotherapy treatment seems to impair muscle strength, quality of life, and fatigue levels [9,10,11,12,13]. However, it is important to highlight that chemotherapy treatments typically involve several separate cycles (e.g., four cycles) [14], and that previous studies have not identified which chemotherapy cycle results in the attenuation of muscle strength, quality of life, and fatigue levels. Klassen et al. [9] examined muscle strength in healthy women and in different cancer treatment groups: a) no chemotherapy treatment, b) initiating chemotherapy treatment (i.e., post first or second cycles), and c) after the chemotherapy treatment. Knee extensor isokinetic strength was lower in the group that completed chemotherapies in comparison to the healthy group. On the other hand, isokinetic strength was not affected in the group that initiated the chemotherapy treatment. Thus, it is unknown if muscle strength, quality of life, and fatigue levels are affected following the third or following the fourth cycle of chemotherapy.

Therefore, evaluating this issue may help professionals who work with this population (e.g., physiotherapists) to design strategies, such as exercise, in order to counteract the side effects of chemotherapy. Exercise has been considered a non-pharmacological strategy for those undergoing breast cancer treatment [3], because it can enhance muscle strength, improve quality of life, and improve fatigue levels [7,15,16]. Thus, this study aimed to evaluate the effects of chemotherapy treatment on muscle strength, quality of life, fatigue, and anxiety in women with breast cancer.

## 2. Methods

### 2.1. Subjects

The participants were separated into a breast cancer treatment group (BCT) and a control group (CNT). Nineteen women who participated in the BCT group (52.2 ± 13.1 years old) were between the third and fourth cycles of chemotherapy treatment, and whose treatment was AC + T (Doxorubicin, Cyclophosphamide + Paclitaxel), and 18 women were in the CNT group (55.8 ± 8.4 years old). The participants’ characteristics are shown in Table 1.

Inclusion criteria for participating in this study included being post-menopausal and not participating in any regular exercise training during the past 6 months. Recommendations of the World Health Organization [17] were followed to determine post-menopausal status. Participants with any osteomioarticular limitations which could compromise performing the study protocol were excluded. Volunteers of the BCT group were selected from the University Hospital, whereas the CNT group was selected from the central region and the city outskirts. Participants were informed about the purpose of the study and possible risks, benefits, and discomforts of the study before signing an informed consent form. The study was approved by the University Institutional Ethics Committee (Protocol: 50717115.4.0000.5083).

**Figure 1:**
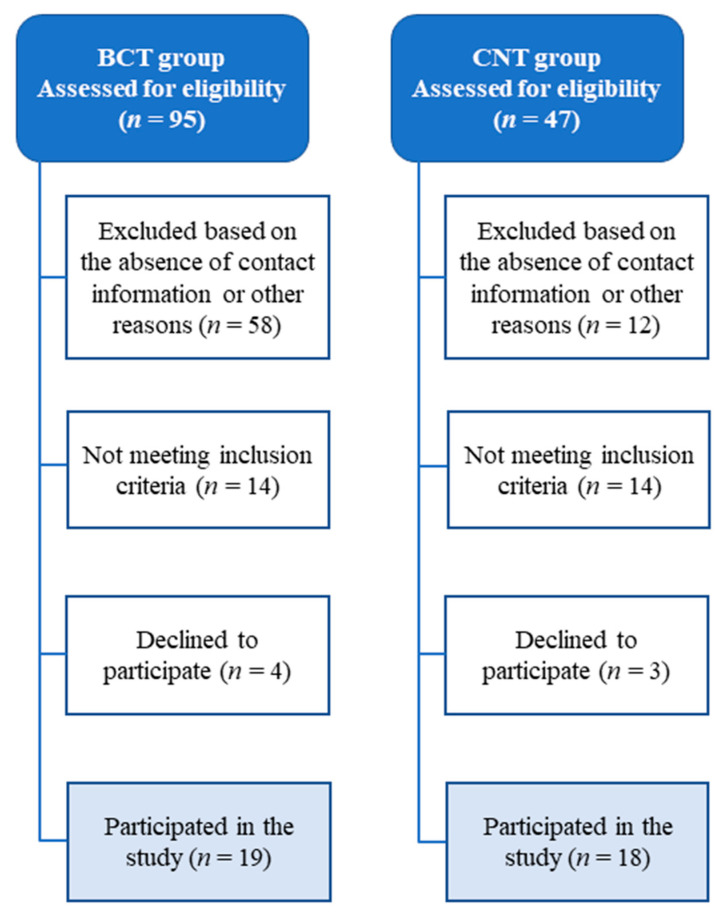
The flow chart of participants enrolled in the study. BCT; breast cancer treatment group. CNT; control group.

The flow chart of participants enrolled in the study is displayed in Figure 1. The BCT group was selected from 95 medical records from the University Hospital. Only 19 BCT volunteers met the inclusion criteria. Forty-seven women were initially recruited for the CNT group; however, only 18 volunteered to participate and met the inclusion criteria. Participants’ characteristics of both the BCT and CNT groups are shown in Table 1. There were no differences in physical characteristics (*p* > 0.05) between groups.

### 2.2. Design

To evaluate the effects of chemotherapy treatment on muscle strength, quality of life, fatigue, and anxiety, participants from both groups attended the laboratory once. During this visit, all participants completed an anamnesis form and completed the anthropometric assessment. Then they answered questions from the fatigue scale, and quality of life and anxiety questionnaires. Thereafter, participants were familiarized with the isometric grip test and an isokinetic knee extension test [18], and three minutes later, participants performed both tests in the same order to assess muscle strength.

### 2.3. Anthropometric Assessment

Body mass was measured using an analog weight scale (Filizola, mod. Personal 7708, Brazil) and height by a stadiometer (Seca, Brazil). Body mass and height were assessed according to the procedures described by Lohman et al. [19].

### 2.4. Physical Activity Level, Fatigue, Quality of Life, and Anxiety

Fatigue levels were assessed by using the Fatigue Scale Questionnaire by Piper et al. [20], which consists of 22 items subdivided into four different subjective dimensions: (1) affective, (2) sensory, (3) cognitive, and (4) behavioral. The total score was calculated from the mean of each item. Each dimension was calculated from the mean of its individual items. Scores can vary from 0 (absence of fatigue) to 10 (severe level of fatigue) with this instrument [21,22].

Quality of life was evaluated using the Short-Form Healthy Survey (SF-36) questionnaire, which is divided into eight domains: (1) functional capacity, (2) physical limitations, (3) pain, (4) general state, (5) vitality, (6) mental health, and (7) social and (8) emotional aspects [18]. Scores can vary from 0 (worse general health) to 100 (better health status) [23].

Anxiety was assessed using the State-Trait Anxiety Inventory (IDATE), an instrument that consists of two separate self-reported scales: (1) anxiety state, and (2) anxiety-trait. Each scale contains 20 statements, in which subjects are required to describe how they generally feel. Overall scores can vary from 20 (mild or low anxiety) to 80 (very-high anxiety or panic levels) [24,25].

### 2.5. Muscle Strength

Muscle strength was assessed by the isometric grip and isokinetic knee extension tests. Both tests were performed for the dominant limb. Isometric grip isometric strength was measured by a hand dynamometer (Camry, model EH101, E.Clear™, Guangdong, China), according to procedures described by the American Society of Hands Therapists [21]. The test consisted of three attempts of 3 to 5 s of maximum voluntary contraction with the elbow flexed at 90°. One min of rest was provided between each attempt [26]. During the test, volunteers remained seated, with their backs supported in a chair and hip and knee joints were maintained at 90°. The attempt with highest isometric strength was considered for statistical analysis.

Knee extension isokinetic strength was measured by an isokinetic dynamometer (Biodex Medical, Inc., Shirley, NY, USA) according to procedures described elsewhere [27]. Three minutes after the isometric grip test, volunteers performed two sets of four unilateral knee extension repetitions at 60°.s^-1^. Two min of rest were given between each set [18]. Three minutes later, participants also carried out one set of 20 repetitions at 180°.s^−1^ [27]. Values for the isokinetic variables were automatically adjusted for gravity with the Biodex Advantage software (Biodex Medical Inc., Shirley, NY, USA). The highest peak torque values for 60°.s^−1^ and 180°.s^−1^ were considered for statistical analysis. Total work for both 60°.s^−1^ and 180°.s^−1^ were also recorded. Additionally, participants received verbal encouragement throughout all tests.

### 2.6. Statistical Analysis

Data are presented as mean and standard deviation. Normal data distributions for physical characteristics, fatigue, quality of life, anxiety, and muscle strength were tested with the Shapiro–Wilk test. An independent *t*-test was used to compare physical characteristics, isometric grip strength, peak torque, and total work between groups. Given that fatigue, quality of life, and anxiety data did not present a normal distribution, the nonparametric Mann–Whitney test was used to analyze these variables. Significance level was set a priori at *p* < 0.05. Additionally, to examine the magnitude effect of chemotherapy treatment, Cohen’s *d* effect sizes were calculated from the differences between group scores divided by the pooled standard deviation [28]. The obtained *d* values were used to define the chemotherapy treatment effect as trivial (*d* < 0.2), small (0.2 ≤ *d* < 0.5), medium (0.5 ≤ *d*< 0.8), and large (*d* ≥ 0.8) [28].

## 3. Results

Quality of life was divided into eight domains (Table 2). Physical limitations, social, and emotional aspects were lower in the BCT group when compared with the CNT group (*p* < 0.05). They presented a large effect size (Table 2). The other domains did not differ between groups (*p* > 0.05). In addition, the effect sizes were medium for vitality and mental health, and small or trivial for the other domains (Table 2).

There were no differences in fatigue and anxiety levels between the BCT and CNT groups (*p* > 0.05, Table 3). Additionally, the effect sizes for affective and cognitive dimensions of fatigue were small, and medium for the other dimensions. A medium effect size was detected for anxiety state, and trivial for anxiety trait (Table 3).

Additionally, isometric grip strength was higher in the CNT group when compared with the BCT group (*p* = 0.048), with an observed medium effect size (Table 4). However, peak torque and total work at both 60°.s^-1^ and 180°.s^-1^ did not differ between the BCT and CNT groups (*p* > 0.05). The effect sizes for the isokinetic tests were all trivial or small (Table 4).

## 4. Discussion

This study aimed to evaluate the effects of chemotherapy treatment on muscle strength, quality of life, anxiety, and fatigue in women with breast cancer. The main results were that three of eight domains related to quality of life, and isometric grip strength, were lower in the BCT group in comparison to the CNT group. These results corroborate previous studies investigating the side effects of chemotherapy treatment [11,29]. They also suggest that quality of life and isometric strength may be reduced in breast cancer patients that had completed three of four cycles of chemotherapy. However, a causal inference is limited since this is a cross-sectional study. Although fatigue and anxiety levels did not differ between groups, medium effect sizes were found for three dimensions of fatigue (i.e., behavior, sensory, and general dimension) and anxiety state.

Chemotherapy treatment has been associated with a compromised functional capacity and reduced quality of life and life expectancy [4]. As a consequence, optimal levels of muscular strength seem to be crucial for women undergoing chemotherapy treatment, since this parameter has been associated with an increased risk of death by cancer [5,30]. Grip strength is known to be a valid and reliable testing procedure. Low levels of grip strength are associated with falls and an increased risk for mortality [31,32]. These assumptions are in agreement with the current results. Oxidative stress, triggered by chemotherapy cycles, has been suggested as a potential mechanism responsible for the side effects of chemotherapy treatment [31,32]. A previous study observed an increased fatigue in women with breast cancer post-adjuvant chemotherapy [33], which was attributed to the use of taxanes, whose side effect is fatigue and pain [34].

Another study evaluated quality of life, fatigue, and anxiety levels after 27 days of chemotherapy treatments in women with breast cancer using the quality of life core questionnaire (EORTC QLQ-C30), Multidimensional Fatigue Inventory (MFI-20) and Hospital Anxiety and Depression Scale (HADS) [35]. These psychometric variables were not affected by the chemotherapy. A possible factor that can explain the divergence between the Cornette et al. [35] results and the present study is the different instruments used to assess the outcome parameters. Another factor that may account for the difference between results is the type of drug used in the chemotherapy treatment. The participants evaluated by Cornette et al. [35] underwent chemotherapeutic treatment with taxanes, whereas in the present study, those in the BCT group were evaluated under the effect of Anthracycline, considered more toxic than taxanes [34,35,36,37]. Thus, Anthracycline can lead to more severe negative effects on the quality of life when compared with taxanes. Further studies are necessary to evaluate if the type of drugs used in chemotherapy treatment can affect quality of life.

The current results also found a difference between groups for isometric grip strength, but not for knee extensors isokinetic strength. It should be noted that the current BCT group had completed three of the four cycles of chemotherapy, whereas Klassen et al. [9] reported a reduced knee extensors isokinetic strength in the group that finished the total chemotherapy treatment plan. Surprisingly, knee extensors isometric strength was not affected by the cancer treatment. Cornette et al. [35] found no alteration in one-repetition maximum knee extensors strength after chemotherapy treatment in women with breast cancer. A recent study also showed no difference in knee extensors isokinetic strength between a healthy group and a group of breast cancer women who completed one to two sessions of chemotherapy [9]. Noteworthy, impaired knee extensors strength was assessed in the group that accomplished chemotherapy treatment. A recent study from our laboratory showed a reduced knee extensors isokinetic strength in a breast cancer survival group (107.6 ± 19.9 N.m) in comparison with a control group (133.4 ± 8 N.m) during a resistance training session [27]. The breast cancer survival group had been on hormone therapy for at least six months. These results suggest that muscle strength impairment by chemotherapy is a phenomenon that may occur throughout the treatment, and it seemed to be dependent on the number of chemotherapy cycles completed.

Additionally, strength performance in cancer patients has been evaluated by different strength tests (e.g., isometric, one-repetition maximum, and isokinetic tests) [9,26,27,32,33,38,39]. Moreover, several joints have been examined (e.g., knee, shoulder, fist) [18]. It is known that these tests evaluate specific aspects of muscle strength [9,26,27,38], and the results may vary according to the examined joint [40]. Thus, future studies should investigate if different strength tests and different joints being examined are confounding factors in evaluating the effects of chemotherapy on muscle strength.

The present study is not without limitation. Functional capacity of participants during daily living activities was not assessed. Another limitation was the lack of evaluation of muscle strength and psychometric parameters throughout the chemotherapy cycles. Moreover, a high number of participants would be useful to evaluate the participants according to their specific neoadjuvant or adjuvant treatment, since surgery can be a factor that affects muscular performance [41].

Therefore, professionals who work with cancer patients should consider the side effects found either following the third or fourth cycles of chemotherapy treatments on isometric grip strength and quality of life in order to design strategies to improve these parameters. A viable option seems to be resistance training, which has been shown to be safe for the current population and may minimize the negative effects of chemotherapy treatment by improving muscle strength and quality of life [42]. Specific recommendations for resistance training prescription for breast cancer patients are available elsewhere [42].

## 5. Conclusions

The study results indicated that completing three of four total cycles of chemotherapy treatment may impair isometric handgrip strength and quality of life in women with breast cancer. Further studies should investigate the effect of neoadjuvant and/or adjuvant treatment in functional performance and psychometric parameters. The effects of drug types used in chemotherapeutic treatment (i.e., taxanes and Anthracycline) should also be examined. Finally, it is suggested to consider the use of longitudinal experimental designs in future studies to assess the side effects of chemotherapy treatment on muscle performance and psychometric variables.

## Figures and Tables

**Table 1:** Physical characteristics of BCT and CNT groups.

Groups	Age (years)	Height (cm)	Weight (kg)
BCT (*n* = 19)	52.2 ± 13.1	160 ± 7	66.8 ± 12.3
CNT (*n* = 18)	55.8 ± 8.4	160 ± 6	69 ± 11.5

BCT; Breast cancer treatment. CNT; Control.

**Table 2:** Quality life levels of BCT and CNT groups.

Domains	BCT Group (*n* = 19)	CNT Group (*n* = 18)	Effect Size	*p-*Value
Functional capacity	67 ± 22	75 ± 21	0.4	0.24
Physical limitations	71 ± 39	32 ± 34	0.9	<0.01
Pain	53 ± 26	62 ± 22	0.4	0.23
General state	59 ± 22	61 ± 19	0.1	0.81
Vitality	58 ± 24	71 ± 20	0.6	0.08
Social aspects	50 ± 31	81 ± 27	0.9	<0.01
Emotional aspects	35 ± 46	87 ± 31	1.1	<0.001
Mental health	75 ± 22	60 ± 25	0.6	0.063

BCT; Breast cancer treatment. CNT; Control group.

**Table 3:** Fatigue index and anxiety levels of BCT and CNT groups.

Dimensions	BCT Group (*n* = 19)	CNT Group (*n* = 18)	Effect Size	*p-*Value
Behavior fatigue	4 ± 3	2 ± 3	0.6	0.08
Affective fatigue	4 ± 4	2 ± 3	0.4	0.18
Sensory fatigue	4 ± 3	2 ± 2	0.6	0.09
Cognitive fatigue	4 ± 3	3 ± 2	0.3	0.34
General fatigue	4 ± 3	2 ± 2	0.6	0.09
Anxiety state	45 ± 12	39 ± 9	0.6	0.08
Anxiety trait	44 ± 13	43 ± 11	0.1	0.92

BCT; Breast cancer treatment. CNT; Control group.

**Table 4:** Muscle strength of the BCT and CNT groups.

	BCT Group (*n* = 19)	CNT Group (*n* = 18)	Effect Size	*p*-Value
Isometric grip strength	23.6 ± 5.6	27.3 ± 4.3	0.7	0.048
Peak torque at 60°.s^−1^ (N.m)	101.1 ± 26.8	101.7 ± 26.9	0.1	0.95
Total work at 60°.s^−1^ (J)	308.4 ± 101.5	325.8 ± 100.9	0.2	0.61
Peak torque at 180°.s^−1^ (N.m)	62.8 ± 13.4	62.5 ± 17	0.1	0.94
Total work at 180°.s^−1^ (J)	971.6 ± 286.2	1004.8 ± 272	0.1	0.72

BCT; Breast cancer treatment. CNT; Control.
